# Effects of Gluten-free Diet on Lipid Profile Risk Factors in Celiac Children

**DOI:** 10.1097/PG9.0000000000000083

**Published:** 2021-06-15

**Authors:** Alessia Angi, Nella Polidori, Marina Cerruto, Francesco Chiarelli, Anna Angelika Mohn

**Affiliations:** From the * Department of Pediatrics, “G. D’Annunzio” University of Chieti, Chieti, Italy; † Department of Pediatrics, Center of Excellence on Aging, “G. D’Annunzio” University of Chieti, Chieti, Italy.

**Keywords:** celiac disease, gluten-free diet, dyslipidemia, atherosclerosis

## Abstract

Many studies raise concerns about the nutritional consequences of gluten-free diet. It has been documented that gluten-free (GF) foods have a higher glycemic index, saturated fats, and lower content of micronutrients determining important health implications. In this retrospective study, we evaluated the change in cardiometabolic risk factors in prepubertal celiac children in remission following different gluten-free diet regimes. Patients using processed GF foods showed a significant increase over time for standard deviation score-total cholesterol, standard deviation score-low density lipoprotein cholesterol, and fasting glycemia. These alterations were not confirmed in patients using naturally GF foods. Therefore, processed GF foods might promote unfavorable alterations of metabolic parameters, especially those associated with increased risk of cardiovascular diseases. Supervision of a dietitian and medical practitioner is recommended to ensure nutritional adequacy and monitoring of cardiovascular risk factors in this population.

**What Is Known**

- Celiac disease is a chronic immune-mediated enteropathy triggered by gluten in genetically predisposed subjects, requiring a strict lifelong gluten-free diet (GFD) for remission.
- Processed gluten-free (GF) foods are higher in fat, sugar, and sodium and lower in protein and dietary fiber contents compared with natural products. However, the GFD’s effects on components of metabolic syndrome, in particularly on lipid profile, are still unclear.

**What Is New**

- Patients do not increase standard deviation score-body mass index with GFD once remission has been achieved.
- Patients consuming processed GF foods show unfavorable lipid profile changes and increased fasting glycemia, which does not occur in patients using naturally GF foods.

To date, the only available treatment for celiac disease (CD) is a strict adherence to a lifelong gluten-free diet (GFD) (1). However, the effects of GFD on cardio-metabolic risk factors currently provide conflicting data. In particular, recent literature regarding the long-term consequences on lipid profile is scarce and includes mainly patients before and after starting GFD. Processed and naturally available gluten-free (GF) products have a different nutritional composition mainly in terms of fats contents, glycemic index (GI) and micronutrients (2–5). To date, it is widely accepted that high levels of saturated fatty acid intake have a negative effect on blood lipid profile (6), including elevation of low density lipoprotein cholesterol (LDL-C), a well-accepted biomarker for risk of cardiovascular disease (CVD). Furthermore, high GI meals result in a rapid increase in blood glucose and insulin levels, which may then lead to obesity, beta cell dysfunction, an increased risk of type 2 diabetes but also dyslipidemia and CVD (7). Giving the importance of prevention of atherosclerosis and CVD, we conducted a retrospective study of celiac children following different GFD regimes for at least 1 year after remission.

## MATERIALS AND METHODS

We retrospectively analyzed data from 15 prepubertal celiac patients, followed by the Gastroenterology Unit of the Department of Paediatrics, University of Chieti, Italy. The diagnosis of CD was defined according to ESPGHAN 2012 guidelines (8).

Inclusion criteria for the study were first evaluation of lipid profile (time_1) assessed between 1 and 3 years of GFD, good compliance at GFD (negative IgA-TG2), second evaluation of lipid profile (time_2) within a maximum of 5 years of GFD. The choice of time_1 has been made to ensure a complete intestinal recovery with normal intestinal absorption for all nutrients, while time_2 has been chosen to avoid a confounding effect of puberty on diet habits and lipid profile. Patients with comorbid conditions potentially interfering with cardiovascular risk factors independently of GFD (type 1 diabetes, thyroid disease, and juvenile idiopathic arthritis) were excluded. Patients were divided into 2 groups: group 1 (8 patients, 4 females and 4 male), children using mainly processed GF products of at least 4 products a day; group 2 (7 patients, 4 females and 3 males): children that prefer naturally occurring GF foods with less than 2 products a day of processed food.

The 15 patients (female 8) were enrolled with a median age at time_1 of 7.23 ± 1.79 years and at time_2 of 8.62 ± 2.02 years. We analyzed anthropometric measures [height, weight, body mass index (BMI), systolic blood pressure, SBP, and diastolic blood pressure, DBP] and laboratoristic values (total cholesterol, TC, low density lipoprotein cholesterol, LDL-C, high density lipoprotein cholesterol, HDL-C, triglycerides, TG, and glycemia).

All included patients had anthropometric measurements and laboratoristic results at both timepoints.

All variables were expressed in terms of Standard Deviation Score (SDS). Wilcoxon test was used to evaluate differences in the study variables between time_1 and time_2. Mann-Whitney test was used to determine differences in the study variables between group 1 and group 2. Two-tailed significance was set to *P* < 0.05.

Ethics approval for this study was not required since it was an audit and it was confined to anonymized and unidentifiable data that are routinely collected at the Gastroenterology Unit of the Department of Paediatrics, University of Chieti, Italy. However, informed consent has been obtained from parents and patients to collect data.

## RESULTS

Anthropometric parameters are shown in Table 1. No differences were found in SDS-BMI between the 2 times of evaluation for the whole group and when analyzing the 2 groups separately. SDS-SBP and SDS-DBP resulted significantly higher at time_2 when compared to time_1 and remained statistically higher for SDS-DBP in children using mainly processed GF products 1.

**TABLE 1.** Clinical characteristics of the study population (n: 15) at time_1 and time_2

	All CD patients (n: 15)			CD patients using processed GF foods (n: 8)			CD patients using naturally GF foods (n: 7)		
	Time_1	Time_2	*P*	Time_1	Time_2	*P*	Time_1	Time_2	*P*
**Age**	7.23 ± 1.79	8.62 ± 2.02	**0.00**	7.14 ± 1.69	8.61 ± 1.85	**0.00**	7.35 ± 2.09	8.63 ± 2.45	**0.00**
**Years of GFD**	2.44 ± 0.42	3.86 ± 0.68	**0.00**	2.34 ± 0.39	3.81 ± 0.74	**0.00**	2.58 ± 0.47	3.95 ± 0.63	**0.00**
**Weight**	27.33 ± 5.08	32.2 ± 7.28	**0.00**	27.44 ± 5.05	32.33 ± 7.35	**0.00**	27.17 ± 5.6	32 ± 7.87	**0.00**
**Height**	123.13 ± 12.85	130.9 ± 14.84	**0.00**	121.22 ± 12.41	129 ± 15.8	**0.00**	126 ± 14.12	133.75 ± 14.16	**0.00**
**SDS-height**	0.08 ± 1.19	0.19 ± 1.26	0.334	–0.18 ± 1.33	–0.03 ± 1.45	**0.28**	0.48 ± 0.91	0.53 ± 0.89	0.81
**BMI**	18.02 ± 2.12	18.73 ± 2.26	**0.009**	18.71 ± 2.45	19.46 ± 2.4	**0.019**	17 ± 0.95	17.65 ± 1.65	0.23
**SDS-BMI**	0.49 ± 1.01	0.42 ± 0.99	0.262	0.71 ± 1.16	0.61 ± 1.14	**0.187**	0.17 ± 0.71	0.14 ± 0.73	0.81
**SBP**	98 ± 6.76	102 ± 7.53	**0.00**	97.2 ± 7.12	101.1 ± 6.51	**0.00**	96.7 ± 4.08	103.3 ± 7.52	**0.03**
**SDS-SBP**	–0.19 ± 1.03	0.26 ± 0.97	**0.01**	–0.25 ± 1.16	0.11 ± 1.02	**0.141**	0.14 ± 0.94	0.34 ± 0.86	0.57
**DBP**	60.67 ± 4.17	64.33 ± 3.71	**0.00**	60 ± 5	65 ± 3.53	**0.00**	61.67 ± 2.58	63.3 ± 4.08	0.17
**SDS-DBP**	–0.025 ± 0.69	0.42 ± 0.62	**0.019**	–0.13 ± 0.79	0.58 ± 0.51	**0.004**	0.13 ± 0.53	0.18 ± 0.74	0.85

A *P* value < 0.05 was considered statistically significant.

BMI = body mass index; CD = celiac disease; DBP = diastolic blood pressure.; GF = gluten free; GFD = gluten-free diet; SBP = systolic blood pressure.

Biochemical value profile are shown in Table 2. Between the 2 timepoints, no significant difference was found in terms of SDS-TC, SDS-HDL-C, SDS-TG, and TG/HDL ratio, whereas SDS-LDL-C and glycemia resulted significantly higher at time_2 when compared with time_1. These changes over time were not confirmed for patients using naturally GF foods, whereas SDS-TC, SDS-LDL-C, and glycemia increased significantly over time for patients using processed GF foods. In fact, at time_2 TC and LDL-C were significantly different between the 2 groups.

**TABLE 2.** Lipid profile and glycemia evaluation in celiac children on GFD, celiac children using processed GF foods, celiac children consuming naturally GF products

	All CD patients			CD patients using processed GF foods			CD patients using naturally GF foods		
	Time 1	Time 2	*P*	Time 1	Time 2	*P*	Time 1	Time 2	*P*
**SDS-TC**	–0.87 ± 0.8	–0.31 ± 1.28	0.067	–0.88 ± 0.73	–0.32 ± 0.85	**0.001**	–0.85 ± 0.97	–1.27 ± 1,25	0.21
**SDS-HDL-C**	0.33 ± 1.44	0.23 ± 0.99	0.74	0.25 ± 1.75	0.27 ± 0.99	0.96	0.46 ± 0.94	0.17 ± 1.09	0.34
**SDS-TG**	–0.13 ± 1.38	–0.01 ± 1.21	0.55	–0.02 ± 1.67	0.07 ± 1.41	0.77	0.36 ± 0.91	–0.15 ± 0.91	0.24
**TG/HDL-C**	1.09 ± 0.48	1.05 ± 0.35	0.72	1.08 ± 0.55	1.1 ± 0.44	0.88	1.13 ± 0.4	0.99 ± 0.12	0.51
**SDS-LDL-C**	–1.08 ± 0.6	–0.51 ± 1.14	**0.017**	–0.93 ± 0.53	–0.11 ± 0.8	0.001*	–1.32 ± 0.68	–1.43 ± 0.95	0.66*
**Glycaemia**	74.4 ± 5.47	79 ± 5.77	**0.014**	74.33 ± 6.65	79 ± 5.81	**0.009** ^†^	74.5 ± 3.61	76.33 ± 6.26	0.29^†^

*CD patients using naturally GF foods lost the statistical significance for the increase of SDS LDL-C.

†CD patients using naturally GF foods lost the statistical significance for the increase of fasting glycemia.

CD = celiac disease; GF = gluten-free; HDL-C = high density lipoprotein cholesterol; LDL-C = low density lipoprotein cholesterol; SDS = standard deviation score; TC = total cholesterol; TG = triglycerides.

## DISCUSSION

Over the last years, the consequences of GF foods on BMI and lipid profile have been analyzed in several studies, which however consider untreated CD children (before and after the start of GFD) and evaluate the positive effects of GFD on BMI and lipid concentrations (9–12). In fact, these studies mainly document that both revert to normal as a consequence of intestinal recovery and improvement in fat absorption.

We instead, excluding the time of diagnosis and including only children in remission documented by negative antibodies (transglutaminase IgA), were interested in the long-term effects of GFD on weight gain and lipid profile. With respect to BMI, no changes were documented in children using processed GF foods neither in children using naturally GF foods indicating that after normalizing of BMI, no further excessive weight gain was obtained with both diet regimens.

Interestingly our study documents that celiac patients consuming mainly processed GF foods show unfavorable changes in metabolic parameters characterized by an increase of TC, LDL-C, and fasting glycemia. These alterations were not detected in children using naturally GF foods. This is particularly worrying as all children are prepubertal, and the unfavorable changes were detected within a short period of observation. These findings are not attributable to further increase of BMI but seem to be associated with the nutritional content of processed GF products characterized by high content in saturated fats and sodium, low levels in micronutrients and a high GI (13) to improve the mouthfeel of these products.

It might, therefore, be argued that after diagnosis, processed GF foods might be helpful in revert to normal lipid profile and weight gain as a consequence of intestinal recovery and improvement in fat adsorption (14). In contrast, on remission phase, the same products might promote alterations of lipid profile, including elevation of TC and LDL-C and increased fasting glycemia, all well-known and accepted biomarkers of CVD (15).

The main limitation of our study is the small sample size. However, the choice of remission phase in prepubertal children with a good compliance to GFD eliminates the confounding factors of hormonal and postmalabsorption induced changes of metabolic parameters. In this context, the findings of statistically significant changes in a small but high-selected population ensure the validity of our data. Furthermore, these findings lay foundation to realize a longitudinal study with a larger sample size that will better define the cardio-metabolic changes related to a long-term GFD.

In conclusion, our findings are of primary importance for infants and young children due to the potential life-long consequences of early diet and suggest that celiac patients need to be accurately supervised by a dietitian who ensures nutritional adequacy of GFD. Naturally, gluten-free foods should be preferred as they are more balanced in terms of lipid composition and micronutrient content as opposed to processed GF products (16–19). These results are in line with previous reports, as in a recent case-control prospective study, Lionetti et al (20) showed that the diet of children with CD was nutritionally less balanced than non-CD with a higher intake of fat and a lower intake of fiber, highlighting the need for dietary supervision. Furthermore, accurate follow-up of these patients for early detection of lipid profile changes is strongly recommended.
